# The Impact of Novel Therapies on Quality-of-Life in Triple-Negative Breast Cancer: A Systematic Review of Clinical Trials

**DOI:** 10.3390/cancers17203307

**Published:** 2025-10-13

**Authors:** Banice Kamau, Maxim Shulimovich, Sinha Samridhi

**Affiliations:** 1 Department of Biomedical Research, St. George’s University, University Centre Grenada, St. George's GE01, Grenada; 2 Department of Hematology and Oncology, The Brooklyn Cancer Center, Brooklyn, NY 11201, USA; 3 Department of Hematology and Oncology, New York Cancer & Blood Specialists, New York, NY 10026, USA

**Keywords:** triple negative breast cancer, immunotherapy, pembrolizumab, atezolizumab, sacituzumab-govitecan, olaparib, talazoparib, quality of life, patient reported outcomes

## Abstract

Background: Triple-negative breast cancer (TNBC) is an aggressive breast cancer subtype characterized by poor prognosis and limited therapeutic options. Chemotherapy regimens are associated with significant adverse effects negatively impacting patients’ quality of life (QoL). This systematic review aims to evaluate and compare QoL outcomes of patients with TNBC receiving novel therapies—including immunotherapy, antibody–drug conjugates, and targeted therapies—versus standard chemotherapy. Methods: We systematically reviewed randomized controlled trials (RCTs) published within the past 15 years, identified through comprehensive searches in PubMed, Google Scholar, Research4Life, and Elicit. Included studies involved FDA-approved novel therapies (pembrolizumab, atezolizumab, sacituzumab-govitecan, olaparib, and talazoparib) administered to TNBC patients, and assessed QoL using validated tools such as EORTC QLQ-C30. Observational studies, case reports, and non-standardized assessments were excluded. Results: Eight RCTs comprising 3929 patients met the inclusion criteria. Sacituzumab govitecan and PARP inhibitors (olaparib and talazoparib) significantly improved QoL, notably delaying deterioration across physical, emotional, and functional domains compared to standard chemotherapy. Conversely, immunotherapies (pembrolizumab, atezolizumab) showed non-significant trends toward QoL improvement, with effects varying by patient subgroup and disease stage. Interpretation was limited by study design differences, inconsistent compliance, and incomplete data reporting. Conclusions: Immunotherapy showed a neutral effect on quality of life, providing neither significant improvement nor additional decline. Olaparib was associated with a delayed deterioration in quality of life, showing a more favorable tolerability profile compared to chemotherapy. Talazoparib leads to clinically meaningful enhancements in quality of life, while sacituzumab govitecan effectively improves patient-reported outcomes relative to standard chemotherapy.

## Simple Summary

Triple-negative breast cancer (TNBC) is an aggressive form of breast cancer with limited treatment options and poor clinical outcomes. Recently, novel therapies, such as immunotherapy (e.g., pembrolizumab), antibody–drug conjugates (e.g., sacituzumab govitecan), and targeted treatments (e.g., PARP inhibitors like olaparib and talazoparib), have been introduced to expand therapeutic options. This systematic review evaluated clinical trials to assess how these novel therapies affect patients’ quality of life (QoL) compared to traditional chemotherapy. Results indicated that some novel therapies, particularly sacituzumab govitecan and PARP inhibitors, showed improvements in patient QoL, delaying deterioration in physical and emotional health. However, immunotherapies showed mixed results with insignificant QoL benefits.

## 1. Introduction

Triple-negative breast cancer (TNBC) represents one of the most aggressive and challenging subtypes of breast cancer, accounting for approximately 15–20% of all breast cancer cases [1,2]. It is characterized by undetectable staining for estrogen (ER) and progesterone (PR) receptors and low or absent expression of HER2/neu, making treatment with endocrine and HER2-targeted therapies less effective. The clinical course of TNBC is often marked by early recurrence, with a higher propensity for visceral and brain metastases [3]. Despite its aggressive nature, TNBC is more frequently diagnosed in younger women and those with germline-associated BRCA mutations, and it tends to be associated with poorer survival outcomes when compared to other breast cancer subtypes [1]. In metastatic settings, the median overall survival (OS) ranges between 8 and 13 months [2]. Given the aggressive behavior and poor prognosis of TNBC, there is a need for improved therapies and comprehensive care strategies that target cancer and enhance the patient’s quality of life.

Health-related quality of life (HRQOL) and patient-reported outcomes (PROs) are measures for evaluating the impact of TNBC therapies beyond survival statistics [4]. While survival rates provide clinical insights, HRQOL and PRO assessments reflect the physical, emotional, and social challenges that patients experience throughout their treatment journey. These measures offer perspectives on the severity of symptoms, treatment side effects, and the overall impact of the disease and its treatment on patients’ daily lives. Incorporating PROs in clinical trials ensures that the patient’s voice is included in understanding therapeutic efficacy and guiding clinicians in balancing treatment benefits with tolerability, especially as TNBC treatments evolve to include newer therapies [4].

The introduction of novel therapies, such as immune checkpoint inhibitors, has marked a significant advancement in treating metastatic TNBC. Our study aims to address the gap in the existing literature by conducting a systematic review comparing the Quality of Life (QoL) outcomes of TNBC patients treated with novel therapies versus chemotherapy. Using data from clinical trials, we seek to determine whether these novel therapies offer a QoL advantage over chemotherapy. The research question is as follows: How do novel therapies compare to chemotherapy in terms of QoL in triple-negative breast cancer patients? Our research conjecture theorizes that novel therapies will lead to better QoL outcomes than chemotherapy. This hypothesis assumes that these therapies have a reduced toxicity profile, resulting in fewer side effects, such as fatigue and nausea, which are commonly associated with chemotherapy [3,5]. The underlying belief is that the lower side-effect burden of novel therapies may enhance physical, emotional, and social well-being, ultimately improving QoL for TNBC patients.

## 2. Materials and Methods

A literature search was conducted in PubMed, Google Scholar, Research4life, and Elicit to identify relevant clinical trial studies that reported on the quality of life (QoL) in triple-negative breast cancer (TNBC) patients treated with novel therapy regimens. Research4life is a platform that provides access to a vast collection of academic and professional resources, including peer-reviewed journals, books, and databases. Elicit is an AI-powered search tool that identifies studies aligned with the research question by scanning large datasets and identifying relevant publications based on keywords, abstracts, and study parameters. It is important to note that neither Research4Life nor Elicit are generative AI tools. Instead, they are advanced research tools designed to locate and retrieve studies aligned with the research question. This approach ensured the inclusion of relevant, high-quality studies while maintaining a systematic and comprehensive literature search process suitable for addressing the objectives of this review.

The following search terms were used in different combinations as queries to identify relevant studies: “immune checkpoint inhibitor,” “immunotherapy,” “pembrolizumab,” “atezolizumab”, “Sacituzumab-govitecan”, “Olaparib”, “Talazoparib”, “quality of life”, “patient-reported outcomes”, and “triple-negative breast cancer.”

The following inclusion criteria was used to select eligible studies: (1) randomized controlled trials (RCTs) in patients with triple-negative breast cancer; (2) Studies that reported on novel therapies for triple-negative breast cancer (3) Studies that reported on Quality of Life (QoL) outcomes using validated QoL instruments (e.g., EORTC QLQ-C30, FACT-B, SF-36, QLQ-BR23, or similar instruments); (4) Peer-reviewed journal articles; (5) Published < 15 years ago.

Our exclusion criteria included the following: (1) observational studies, case reports or editorials; (2) RCTs involving other breast cancer subtypes; (3) ongoing studies without published results at the time of the literature search; (4) Studies that did not assess Quality of Life as a primary or secondary outcome; (5) Studies that used non-validated or non-standardized QoL assessment tools.

Of note, the IMpassion trials evaluated atezolizumab in combination with nab-paclitaxel or paclitaxel. However, subsequent confirmatory data did not support continued regulatory approval for atezolizumab, and these regimens have since been removed from the National Comprehensive Cancer Network (NCCN) guidelines and American Society of Clinical Oncology (ASCO) recommendations. Pembrolizumab, in combination with chemotherapy, has subsequently emerged as the preferred first-line immunotherapy for PD-L1-positive metastatic TNBC, as supported by the phase III KEYNOTE-355 trial, which demonstrated significant improvements in both overall survival and progression-free survival [6]. Although no longer part of standard clinical practice, the IMpassion trials remain relevant to the literature because they provide well-characterized HRQoL data of patient experiences with immunotherapy in TNBC. For this reason, they were included in this review to contribute to a more comprehensive understanding of health-related quality of life (HRQoL).

We extracted the following information from each study: the name of the clinical trial, year of publication, type of therapy used, study population (e.g., stage of the cancer, global health status), patient number in both control arm and experimental arm, QoL instrument used, Completion and compliance rates, and, when provided, QoL domain-specific scores, changes over time, Time to Deterioration (TTD), and Time to Improvement (TTI) when provided.

All the studies reported completion and compliance rates. Collectively, the completion rate was defined as the percentage of patients who completed one or more scores or items in the PRO assessment questionnaire at each time point over the number of patients randomized. The compliance rate was defined as the percentage of patients who completed one or more scores or items at each time point over the number of patients who were expected to complete the PRO assessment questionnaire at each time point. Time to Deterioration (TTD) was defined as the time to the first onset of a 10 or more point decrease from baseline score, sustained for two or more consecutive cycles. The remaining articles defined TTD as the time to the first onset of a 10 or more point decrease from baseline score, while Time to Improvement (TTI) was defined as the time to the first onset of a 10 or more point increase from baseline.

First-line systemic therapy for advanced triple-negative breast cancer (TNBC) is stratified based on tumor PD-L1 expression and modulated by the disease-free interval following prior adjuvant treatment. In patients with previously untreated, locally recurrent, unresectable or metastatic TNBC exhibiting PD-L1 positivity—defined by a combined positive score (CPS) ≥10—pembrolizumab in combination with chemotherapy has demonstrated a significant clinical benefit and is considered the preferred therapeutic approach, as established in the KEYNOTE-355 trial [7].

Health-Related Quality of Life (HRQoL) represents a distinct domain within the broader framework of Patient-Reported Outcomes (PROs), specifically addressing the impact of disease and its treatment on an individual’s physical, psychological, and social functioning. PROs are reports provided directly by patients regarding their health condition, symptoms, and treatment experiences, without interpretation by clinicians. These outcomes encompass a wide spectrum of domains, including symptom burden (e.g., fatigue, pain), treatment satisfaction, medication adherence, and functional capacity. While PROs are frequently utilized in regulatory submissions, safety monitoring, and comparative effectiveness research, HRQoL assessments are more commonly applied in the contexts of health economics, survivorship research, and supportive care evaluation. Instruments such as the PRO-CTCAE and Brief Pain Inventory are typically employed to capture general PRO data, whereas validated tools like the EORTC QLQ-C30, FACT-G, and EQ-5D are commonly used to measure HRQoL. Collectively, these measures provide critical insights into the patient perspective and the real-world impact of therapeutic interventions beyond conventional clinical endpoints [8,9].

One of the most widely used QoL tools in cancer research is the EORTC QLQ-C30, a general cancer QoL questionnaire that evaluates multiple domains. The EORTC QLQ-BR23 is a breast cancer-specific module commonly used alongside the QLQ-C30 to assess issues directly related to breast cancer, such as body image, arm symptoms, breast symptoms, and side effects from treatment [8,9]. This combination provides an in-depth understanding of how breast cancer, including TNBC, affects a patient’s physical appearance and emotional state and the more general aspects of well-being.

Another tool is the Functional Assessment of Cancer Therapy—General (FACT-G), which measures functional, social/family, emotional, and physical well-being. When used with the FACT-B module, it addresses issues unique to breast cancer patients, such as concerns about body image and sexual functioning [10]. The EQ-5D-3L is a standardized instrument for measuring health-related quality of life (HRQoL). The EuroQol Group developed it as a generic QoL measure that can be applied across different diseases and treatments [11]. It captures a patient’s overall health status from their perspective. The EQ-5D-3L also includes a Visual Analogue Scale (VAS), where patients rate their overall health from 0 (worst imaginable health) to 100 (best imaginable health). This tool is used in clinical trials and health economics evaluations to provide a summary index value for cost-utility analyses [12]. When used in conjunction, these tools help provide a holistic view of how TNBC patients experience their disease and treatment [13].

The EORTC QLQ-C30 includes five functional domains—physical, role, cognitive, emotional, and social functioning—and a global health status/quality-of-life scale, which together assesses a patient’s level of daily functioning and overall well-being [13]. Additionally, the QLQ-C30 features three multi-item symptom scales (fatigue, pain, and nausea/vomiting) and six single-item scales to address other common issues (such as insomnia, appetite loss, constipation, diarrhea, dyspnea, and financial difficulties) that may affect quality of life [8,9]. The questionnaire uses a 0–100 scale for scoring, with higher scores on the functional and global QoL scales indicating better functioning or health status. In comparison, higher scores on the symptom scales indicate greater severity of symptoms [8,9]. Various QoL tools were used in clinical trials reviewed, including the EORTC QLQ-C30, FACT-G, and EQ-5D-3L. Therefore, to maintain consistency, we report on the results from the EORTC QLQ-C30 questionnaire.

This systematic review was not prospectively registered in PROSPERO because data collection and synthesis had been completed prior to the registration process.

## 3. Results

**Figure 1:**
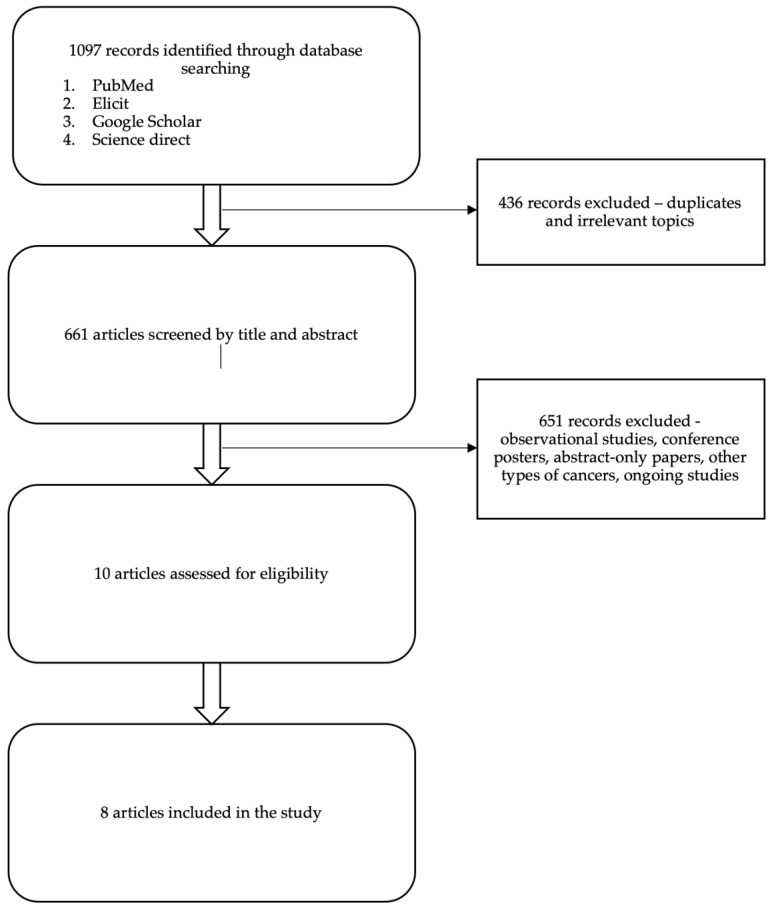
PRISMA flow diagram summarizing the data collection and refinement process.

Eight Randomized Control Trials fulfilled the eligibility criteria (Table 1). Five trials reported on immunotherapy-based regimens (Pembrolizumab and Atezolizumab), one study reported on ADC (Sacituzumab-govitecan), and two studies reported on PARP inhibitors (Olaparib and Talazoparib). The search progress is shown in Figure 1. Altogether, 3929 patients from the final selected studies were included in this review, of whom 2365 received novel therapies and 1564 received standard therapies (Table 1 and Table 2).

For consistency and cohesion in the results, we will report item scores from the EORTC QLQ-C30 questionnaire, which assesses Global Health Status (GHS/QoL), functioning, and symptomatology (Table 3). This tool is widely recognized and was used in all the trials included in this review.

### 3.1. Early-Stage TNBC Novel Therapies

The IMpassion031 trial investigated the addition of atezolizumab to neoadjuvant chemotherapy in patients with early-stage triple-negative breast cancer (stage II/III). The primary focus was on the neoadjuvant phase, with additional observations from the adjuvant phase for context. 333 patients were selected and randomized 1:1 for the PRO evaluation. Of these, 161 patients in the atezolizumab-chemotherapy arm and 167 patients in the placebo-chemotherapy arm were evaluable for PRO using the EORTC QLQ-C30 tool. High compliance rates were reported in both study arms, with assessment tool completion rates of above 88% in the neoadjuvant phase, greater than 88% in the adjuvant phase, and above 85% after treatment discontinuation. The primary endpoints focused on physical and role functioning and HRQoL, while treatment-related symptoms were reported as exploratory PRO endpoints [14].

The KEYNOTE-522 trial evaluated the use of pembrolizumab combined with chemotherapy in the neoadjuvant phase, followed by adjuvant pembrolizumab for early-stage high-risk TNBC, with a focus on both the neoadjuvant and adjuvant phases. 1174 patients were enrolled in the PRO evaluation, with 784 randomized to neoadjuvant pembrolizumab or placebo, and later, 584 patients received adjuvant pembrolizumab. All patients were newly diagnosed, treatment-naive, and had an ECOG performance status of 0 or 1. The PRO assessment tools included the EORTC QLQ-C30, QLQ-BR23 (measuring prespecified secondary endpoints), and the EQ-5D (measuring secondary objectives). Completion and compliance rates at baseline were above 90%. However, these decreased significantly from Cycle 5 (week 21) in the neoadjuvant phase and Cycle 6 (week 40) in the adjuvant phase, with rates ranging from 60% to 80%. As a result, the adjuvant phase data reflect outcomes from 24 weeks of pembrolizumab therapy alone [15].

#### 3.1.1. Baseline

In the IMpassion031 trial, the baseline mean values for physical functioning, role functioning, and Global Health Status were reported as 90.0, 89.4, and 79.2, respectively, in the atezolizumab-chemotherapy arm.

In the KEYNOTE-522 trial, the baseline mean values for Global Health Status, physical and emotional functioning in the neoadjuvant phase were 77.08, 91.89, and 76.14, respectively. In the adjuvant phase, the baseline mean values were 73.82, 83.30, and 81.75, respectively.

#### 3.1.2. Global Health Status (GHS/QOL)

The IMpassion031 trial reported a clinically meaningful decline in GHS/QOL from Cycle 3 to Cycle 5 (week 3 to week 9), which improved by Cycle 6 (week 12).

In the KEYNOTE-522 trial, GHS/QOL decreased from baseline to Cycle 5 (week 21) in both treatment arms in the neoadjuvant phase. In the adjuvant phase, the score improved from baseline to week 24 in both treatment groups, with a Least Squares mean score difference of −0.41 (95% CI −2.60 to 1.77).

#### 3.1.3. Physical Functioning

In the IMpassion031 trial, patients in the neoadjuvant phase who received atezolizumab-chemotherapy experienced clinically meaningful deterioration in physical functioning at the start of Cycle 3, which was sustained through Cycle 5 (week 3 to week 9). In the adjuvant phase (Cycles 6–16, weeks 12–42), patients experienced gradual stability in physical function.

The KEYNOTE-522 trial reported decreased physical functioning from baseline to Cycle 5 (week 21) in both treatment arms in the neoadjuvant phase. The bar graph presented showed a greater negative impact on the pembrolizumab-chemotherapy arm compared to the placebo-chemotherapy arm. In the adjuvant phase, the score improved from baseline to week 24 in both treatment groups, with a Least Squares mean score difference of −1.57 (95% CI −3.36 to 0.21).

#### 3.1.4. Role Functioning

In the IMpassion031 trial, clinical deterioration in role functioning began in Cycle 2 (week 2) and was sustained through Cycle 9 (week 21), where values stabilized. The graphs showed a greater negative impact on role functioning compared to the placebo arm in both the neoadjuvant and adjuvant phases.

The KEYNOTE-522 trial reported decreased role functioning from baseline to Cycle 5 (week 21) in both treatment arms in the neoadjuvant phase. As shown in the bar graph, role functioning had a greater negative impact on the pembrolizumab-chemotherapy arm compared to the placebo-chemotherapy arm. In the adjuvant phase, the role functioning score improved from baseline to week 24 in both treatment groups.

#### 3.1.5. Emotional, Cognitive, and Social Functioning

In the IMpassion031 trial, social functioning showed clinically meaningful worsening in the neoadjuvant phase, with gradual improvement in the adjuvant phase. However, the mean changes from baseline remained lower in the adjuvant phase compared to the placebo-chemotherapy arm. Emotional functioning remained stable in both phases of treatment (Supplementary File).

In the neoadjuvant phase of the KEYNOTE-522 trial, emotional and cognitive functioning decreased from baseline to Cycle 5 (week 21) in both treatment arms. The bar graph showed similar declines between the treatment arms. Social functioning declined more in the pembrolizumab-chemotherapy arm compared to the placebo-chemotherapy arm.

In the adjuvant phase, the KEYNOTE-522 trial reported decreased emotional functioning (81.75 to 79.62) in the pembrolizumab arm but no change in the placebo-chemotherapy arm. The between-group difference was ‒0.60 (95% CI –2.99 to 1.79). Social functioning improved from baseline to week 24, while cognitive functioning remained unchanged.

#### 3.1.6. Treatment-Related Symptom Items

The IMpassion031 trial reported clinically meaningful worsening of symptoms in the neoadjuvant phase, with pain peaking at Cycle 4 (week 5), and most treatment-related symptoms (fatigue, diarrhea, nausea, vomiting) peaking at Cycle 5 (week 9). Insomnia showed minimal mean changes in the adjuvant phase compared to the placebo arm.

In the adjuvant phase, the study reported improvement in treatment-related symptoms, with values approaching baseline levels, except for fatigue, which remained elevated. Grade 3 or 4 treatment-related adverse events occurred in >10% of patients, including neutropenia, decreased neutrophil count, and febrile neutropenia.

The KEYNOTE-522 trial reported worsening symptoms of fatigue, nausea, vomiting, pain, dyspnea, appetite loss, constipation, and financial difficulties from baseline to week 21 in both treatment groups. Diarrhea and insomnia worsened in the pembrolizumab-chemotherapy arm. Fatigue, appetite loss, constipation, and diarrhea had a greater negative impact on the pembrolizumab-chemotherapy arm compared to the placebo-chemotherapy arm, as shown in the bar graph.

In the adjuvant phase, the KEYNOTE-522 trial reported improved pain, insomnia, and appetite loss in both treatment groups from baseline to week 24. Nausea, vomiting, dyspnea, constipation, and diarrhea remained unchanged in both treatment groups. Financial difficulties were unchanged in the pembrolizumab arm but decreased in the placebo arm. The bar graph showed better improvements in appetite loss and financial difficulties in the placebo arm compared to pembrolizumab. Fatigue worsened in the pembrolizumab arm and remained unchanged in the placebo group (Table S1).

### 3.2. Metastatic Stage TNBC

The IMpassion130 trial evaluated atezolizumab combined with nab-paclitaxel (A + nP) as a first-line treatment for metastatic triple-negative breast cancer (mTNBC). Patients had no prior treatment for mTNBC and were stratified by PD-L1 status, liver metastases, and prior taxane use. In the PRO evaluation study, patients were randomized 1:1 to measure prespecified endpoints of Global Health Status (GHS) and Functioning. Key secondary endpoints included Time to Deterioration (TTD) in physical, role, cognitive functioning, and disease or treatment-related symptoms. The study reported high completion rates (>80%) for the EORTC QLQ-C30 assessment tool in both treatment arms through cycle 20, but completion rates declined significantly during the follow-up period, ranging from 17% to 41% [16].

The KEYNOTE-119 trial compared Pembrolizumab (200 mg IV every 3 weeks for up to 2 years) with chemotherapy as a second- or third-line metastatic TNBC (mTNBC) treatment. Participants had received 1–2 prior systemic treatments for metastatic breast cancer, with documented disease progression after the most recent therapy. Patients were randomized 1:1 to receive pembrolizumab or the physician’s choice of chemotherapy. The PRO evaluable study focused on patients with PD-L1-positive tumors (>10). Prespecified exploratory endpoints in the EORTC QLQ-C30 tool included GHS/QOL, physical functioning, treatment-related symptoms, and TTD in GHS/QOL, physical functioning, nausea, vomiting, and diarrhea. The primary analysis time point was defined as when compliance and completion rates were ~60% and ~80%, respectively, which was reported at week 6. Beyond week 6, completion rates in both arms significantly declined, falling below 50% and as low as 7%. By Week 35, the number of eligible patients expected to complete the PRO was significantly reduced, with 14 in the pembrolizumab group and 9 in the chemotherapy arm [17].

The KEYNOTE-355 trial evaluated Pembrolizumab (200 mg every 3 weeks for 35 cycles—up to 2 years) combined with chemotherapy regimens (nab-paclitaxel, paclitaxel, or gemcitabine plus carboplatin) for patients with advanced or metastatic TNBC (mTNBC). Participants included patients with locally recurrent inoperable or metastatic disease, untreated in the metastatic setting, or with de novo metastasis or recurrence after completing curative treatment 6 months prior. Patients were randomized 2:1 to receive pembrolizumab + chemotherapy or placebo + chemotherapy. The PRO evaluable study focused on patients with PD-L1-positive tumors (>10). Prespecified secondary endpoints in the EORTC QLQ-C30 tool included mean changes in GHS/QOL, physical functioning, and TTD in GHS/QOL, emotional functioning, and physical functioning. The primary analysis time point, where compliance and completion rates were approximately 60% and 80%, was on week 15. Completion and compliance rates were 77% and 87% in the pembrolizumab-chemotherapy arm vs. 70% and 81.4% in the chemotherapy arm. Completion rates declined by week 51 to 35.5% in the pembrolizumab arm and 24% in the chemotherapy arm, with compliance rates remaining >80% for both arms [18].

The ASCENT trial evaluated Sacituzumab govitecan (SG), an antibody–drug conjugate targeting Trop-2, compared to Treatment of Physician’s Choice (TPC), which included standard chemotherapy regimens (capecitabine, eribulin, vinorelbine, or gemcitabine) in patients with refractory or relapsed, unresectable, locally advanced metastatic TNBC (mTNBC) who had received two or more prior systemic therapies. Participants were predominantly adult females with a mean age of 53.8 years in the SG arm and 55.5 years in the chemotherapy arm. Most of the population was White (83% in SG and 76% in TPC). Patients were randomized 1:1 to receive SG or chemotherapy. The primary endpoints in the HRQoL domains were GHS/QOL, physical functioning, role functioning, fatigue, and pain. Secondary HRQoL domains included emotional, cognitive, and social functioning, dyspnea, insomnia, nausea and vomiting, constipation, diarrhea, and financial difficulties. TTD and Time to Improvement (TTI) were also assessed. Mean changes from baseline were reported for data collected in cycle 6, day 1 (week 15). Completion rates were steady at >90% in both treatment arms, but compliance rates significantly reduced in both arms, with a greater reduction in the chemotherapy arm (~55% in cycle 6 vs. ~50% in cycle 3) [19].

The OlympiAD trial compared Olaparib, a PARP inhibitor, with chemotherapy in women with HER2-negative (triple-negative or hormone receptor-positive) metastatic breast cancer with germline BRCA mutations (gBRCAm) who had received two or more prior systemic therapies. Patients were randomized 2:1 to Olaparib (300 mg twice daily) or chemotherapy. Prespecified endpoints included GHS/QOL, functional scales, and TTD. Compliance and completion rates at baseline were >95%, but completion rates declined significantly to 47.3% by week 36. Compliance rates remained high throughout the study period, approximately >84% [20].

The EMBRACA trial compared Talazoparib, a PARP inhibitor, with standard chemotherapy for HER2-negative metastatic breast cancer patients with a germline-associated BRCA1/2 mutation. Patients were randomized 2:1 to Talazoparib (1 mg daily) or chemotherapy. Prespecified endpoints were GHS/QOL, functional subscales, symptom scales, and TTD. Compliance and completion rates from baseline to cycle 13 (week 36) were >81% in the Talazoparib arm and >73% in the chemotherapy arm. However, the number of patients remaining at this point was 107 (37.3%) in the Talazoparib arm vs. 19 (13.2%) in the chemotherapy arm [21].

#### 3.2.1. Baseline

In the IMpassion130 trial, the baseline mean values for Global Health Status (GHS), physical functioning, role functioning, and cognitive functioning in the atezolizumab-chemotherapy arm were reported as 66, 80, 73, and 83, respectively.

In the KEYNOTE-119 trial, the baseline GHS/QoL score in the PD-L1 CPS > 10 population was 68.6.

In the KEYNOTE-355 trial, the baseline mean values for GHS/QoL, physical and emotional functioning were 67.54, 73.34, and 81.56, respectively.

In the ASCENT trial, the baseline mean values for the primary focused domains (GHS/QoL, physical functioning, role functioning, fatigue, and pain) were reported as 63.2, 74.9, 69.6, 38.2, and 36.2. The baseline mean values for secondary focused domains (emotional functioning, cognitive functioning, social functioning, nausea/vomiting, dyspnea, insomnia, appetite loss, constipation, diarrhea, and financial difficulties) were 72.1, 82.5, 70.6, 7.6, 24.7, 31.6, 19.2, 16.6, 7.4, 27.2, and 76.0, respectively. Of note, mean baseline scores were worse in both treatment arms, but worse for TPC vs. SG in GHS/QoL and insomnia, while the baseline score for financial difficulties was worse in SG vs. TPC.

The OlympiAD trial’s baseline mean value for GHS/QoL in the Olaparib arm was 63.2.

In the EMBRACA trial, baseline mean values for GHS/QoL were similar in both treatment arms, reported as approximately 61.9. Additionally, baseline mean values for physical, role, emotional, cognitive, and social functioning were reported as 78.5, 73.5, 67.2, 82.6, and 72.8, respectively. Baseline mean values for fatigue, nausea/vomiting, pain, dyspnea, insomnia, appetite loss, constipation, and diarrhea were 36.6, 9.5, 33.2, 18.7, 34.4, 20.7, 18.1, and 5.5, respectively.

#### 3.2.2. Global Health Status (GHS/QoL)

In the IMpassion130 trial, the graph presented showed comparable mean values between the arms from approximately Cycle 12 to Cycle 30, with the atezolizumab + nab-paclitaxel arm showing higher scores than the placebo-chemotherapy arm. This was reflected in both the ITT and the PD-L1 IC+ population.

In the KEYNOTE-119 trial, the mean score change at week 6 in the PD-L1 population (CPS > 10) was reported to be ‒0.49 (95% CI ‒4.62 to 3.64) in the pembrolizumab arm vs. ‒4.70 (95% CI ‒8.95 to ‒0.46) in the chemotherapy arm.

In the KEYNOTE-355 trial, the change in the mean score at week 15 was ‒2.69 (95% CI = ‒5.86 to 0.48) in the pembrolizumab plus chemotherapy arm, with an in-between group difference of ‒1.81 (95% CI = ‒6.92 to 3.30).

In the ASCENT trial, a statistically significant superiority and clinically meaningful difference in GHS/QoL were reported for SG vs. TPC

In the OlympiAD trial, clinically significant improvement from baseline in GHS/QoL for Olaparib was noted, with a difference of 7.5 (95% CI 2.48–12.44, *p* = 0.0035), which was sustained in subsequent visits.

In the EMBRACA trial, a statistically significant improvement in GHS/QoL was reported in the Talazoparib arm vs. the chemotherapy arm (3.0, 95% CI 1.2–4.8).

##### Time to Deterioration (TTD) in Global Health Status (GHS/QoL)

In the IMpassion130 trial, no significant deterioration in GHS/HRQoL was reported during the treatment period in both arms. The TTD in the atezolizumab–nab-paclitaxel arm was 8.3 months, while in the placebo arm, TTD was 8.0 months (HR 0.97, 95% CI 0.80–1.18, *p* = 0.77). However, meaningful comparisons between the groups were limited due to fewer remaining patients on treatment past Cycle 7. The study also reported declines during the follow-up period after treatment discontinuation.

In the KEYNOTE-119 trial, the median TTD was longer in the pembrolizumab arm, reported to be 4.3 months (95% CI 3.4–5.6) vs. 1.7 months in the chemotherapy arm (95% CI 1.4–3.5).

In the KEYNOTE-355 trial, the median TTD in the pembrolizumab-chemotherapy arm was 5.8 months (95% CI 3.1 to 8.1) vs. 5.6 months (95% CI 3.7 to 9.7).

In the ASCENT trial, the median TTD was similar in both arms (SG vs. TPC) at 14.1 weeks vs. 15.1 weeks, HR = 0.87 (95% CI 0.7 to 1.07, *p* = 0.18).

In the OlympiAD trial, the median TTD was not reached in the Olaparib arm, whereas the chemotherapy arm showed a TTD of 15.3 months (HR 0.44, 95% CI 0.25–0.77, *p* = 0.004). Additionally, more patients in the Olaparib arm improved the best overall response rates for GHS/QoL. Global scores at 6 months were reduced in more patients in the chemotherapy arm vs. the Olaparib arm (38.8% vs. 18.5%) and at 12 months (46.5% vs. 36.0%).

In the EMBRACA trial, a statistically significantly greater delay in TTD was observed in the Talazoparib arm (24.3 months) vs. chemotherapy (6.3 months), HR of 0.376 (95% CI 0.26–0.5, *p* < 0.0001).

#### 3.2.3. Physical Functioning

In the IMpassion130 trial, mean values were comparable between treatment arms from approximately Cycle 7 to discontinuation. Beyond Cycle 35, the atezolizumab + nab-paclitaxel arm showed higher scores when compared to the placebo-chemotherapy arm, reflected in both the ITT and PD-L1 IC+ populations.

In the KEYNOTE-119 trial, the mean values for physical functioning were unchanged from baseline to week 6, while a decline in function was reported in the chemotherapy arm.

In the KEYNOTE-355 trial, the change in the mean score at week 15 was ‒6.76 (95% CI = ‒9.89 to ‒3.62) with an in-between group difference of ‒1.05 (95% CI = ‒6.59 to 4.50), showing no significant difference between the arms.

In the ASCENT trial, SG was superior and reported a statistically significant and clinically meaningful difference in physical functioning compared to TPC.

In the OlympiAD trial, more patients in the Olaparib treatment arm experienced improvement in the functional subscales compared to the chemotherapy arm.

In the EMBRACA trial, a statistically significant improvement in physical functioning was reported in the Talazoparib arm vs. the chemotherapy arm (2.9, 95% CI 0.9–4.9).

##### Time to Deterioration (TTD) in Physical Functioning

In the IMpassion130 trial, no significant deterioration in physical functioning was reported during the treatment period in both arms. The TTD in the atezolizumab–nab-paclitaxel arm was 6.1 months vs. 7.4 months in the placebo arm (HR 1.04, 95% CI 0.86–1.26, *p* = 0.69). Declines were noted during the follow-up period after treatment discontinuation.

In the KEYNOTE-119 trial, the median TTD was longer in the pembrolizumab arm (4.3 months, 95% CI 3.4–10.6) than in the chemotherapy arm (3.4 months, 95% CI 1.6–5.8).

In the KEYNOTE-355 trial, the median TTD in the pembrolizumab-chemotherapy arm was 5.6 months (95% CI 4.0–6.9) vs. 5.8 months (95% CI 3.7–14.5).

In the ASCENT trial, the median TTD was longer in SG vs. TPC (22.1 weeks vs. 12.1 weeks, HR = 0.61, 95% CI 0.49–0.75, *p* < 0.001).

In the OlympiAD trial, TTD was 22.1 months in the Olaparib arm vs. 12.9 months in the chemotherapy arm.

In the EMBRACA trial, a significantly delayed TTD in the Talazoparib arm was observed (28.4 months vs. 7.6 months in the chemotherapy arm), with an HR of 0.30 (95% CI 0.20–0.46, *p* < 0.0001).

#### 3.2.4. Role Functioning

In the IMpassion130 trial, mean values were comparable between the treatment arms from approximately Cycle 7 to discontinuation. Beyond Cycle 35, the atezolizumab + nab-paclitaxel arm showed higher scores than the placebo-chemotherapy arm, which was reflected in both the ITT and the PD-L1 IC+ population.

In the KEYNOTE-355 trial, the mean score decreased in both arms from baseline to week 15. However, as shown in the bar graph, the decline was less severe in the pembrolizumab arm.

In the OlympiAD trial, clinically meaningful worsening was observed at cycle 2 (week 6).

##### Time to Deterioration (TTD) in Role Functioning

In the IMpassion130 trial, the PD-L1 IC+ population showed a longer median TTD in the atezolizumab–nab-paclitaxel arm, where the TTD was reported to be 5.9 months, while in the placebo arm, the TTD was 6.8 months (HR 1.01, 95% CI 0.83–1.22, *p* = 0.93). Of note was that the study reported a decline during the follow-up period when patients discontinued the study treatment. While no clinically meaningful deterioration was observed between treatment arms during the trial, patients experienced declines in functioning and HRQoL during the follow-up phase, likely due to disease progression or second-line therapy toxicities.

In the ASCENT trial, the median TTD was longer in the SG arm vs. TPC (11.4 weeks vs. 7.1 weeks, HR = 0.70, 95% CI 0.56 to 0.86, *p* < 0.001).

In the OlympiAD trial, the TTD was 22.1 months in the Olaparib arm vs. 8 months in the chemotherapy arm.

In the EMBRACA trial, a significantly greater delay in TTD was observed in the Talazoparib arm (20.5 months) vs. chemotherapy (5.6 months), with an HR of 0.36 (95% CI 0.25–0.52; *p* < 0.0001).

#### 3.2.5. Emotional Functioning

In the KEYNOTE-119 trial, the mean values for emotional functioning remained unchanged from baseline to week 6, while a decline in function was reported in the chemotherapy arm.

In the KEYNOTE-355 trial, the change in the mean score at week 15 was ‒0.75 (95% CI = ‒3.92 to 2.43), with an in-between group difference of ‒1.43 (95% CI = ‒7.03 to 4.16).

In the ASCENT trial, statistically significant superiority and clinically meaningful differences in emotional functioning were reported for SG vs. TPC.

In the EMBRACA trial, a statistically significant improvement in emotional functioning was reported in the Talazoparib arm vs. the chemotherapy arm (6.1, 95% CI 3.8–8.4).

##### Time to Deterioration (TTD) in Emotional Functioning

In the KEYNOTE-355 trial, the median TTD in the pembrolizumab-chemotherapy arm was 9.3 months (95% CI 6.4 to 11.7) vs. 15.3 months (95% CI 5.6 to Not Reached).

In the ASCENT trial, the median TTD was longer in SG vs. TPC.

In the OlympiAD trial, the TTD was not reached in the Olaparib arm, compared to 19.7 months in the chemotherapy arm.

In the EMBRACA trial, a significantly delayed TTD was observed in the Talazoparib arm (31.5 months) vs. chemotherapy (9.5 months), with an HR of 0.24 (95% CI 0.15–0.38; *p* < 0.0001).

#### 3.2.6. Cognitive Functioning

In the IMpassion130 trial, no difference was reported in the mean values, which were comparable between treatment arms from approximately Cycle 12 to discontinuation. Beyond Cycle 35, the atezolizumab + nab-paclitaxel arm scored higher than the placebo-chemotherapy arm, reflected in the ITT and PD-L1 IC+ populations.

In the KEYNOTE-119 trial, the mean values were unchanged from baseline to week 6 in cognitive functioning, while a decline in function was reported in the chemotherapy arm.

In the KEYNOTE-355 trial, the mean score decreased in both arms from baseline to week 15.

##### Time to Deterioration (TTD) in Cognitive Functioning

In the IMpassion130 trial, the TTD was observed to be 9.0 months in the atezolizumab–nab-paclitaxel arm and 7.5 months in the placebo arm (HR 0.93, 95% CI 0.76–1.14, *p* = 0.49).

In the OlympiAD trial, the TTD was 16.6 months in the Olaparib arm vs. 10.0 months in the chemotherapy arm.

In the EMBRACA trial, a significantly delayed TTD in the Talazoparib arm was observed (25.6 months) vs. chemotherapy (7.9 months), with an HR of 0.39 (95% CI 0.26–0.58; *p* < 0.0001).

#### 3.2.7. Social Functioning

In the KEYNOTE-355 trial, the mean score decreased in both arms from baseline to week 15. However, as shown in the bar graph, the decline was less severe in the pembrolizumab arm.

In the ASCENT trial, the median TTD was longer in SG vs. TPC

In the OlympiAD trial, the TTD was observed to be not reached in the Olaparib arm vs. 12.5 months in the chemotherapy arm.

In the EMBRACA trial, a significantly delayed TTD was observed in the Talazoparib arm (17.3 months) vs. chemotherapy (7.5 months), with an HR of 0.43 (95% CI 0.30–0.63; *p* < 0.0001) (Tables S2 and S3).

#### 3.2.8. Treatment-Related Symptom Items

In the IMpassion130 trial, mean values for fatigue, diarrhea, and nausea/vomiting were reported not to have changed significantly from baseline but were comparable between treatment arms. However, the mean change at Cycle 7 for symptom scores showed worsening from baseline, with fatigue (4.6), diarrhea (5.3), and nausea/vomiting (0.9). The trial also reported common side effects such as alopecia, fatigue, nausea, and diarrhea.

In the KEYNOTE-119 trial, the mean values for fatigue, nausea/vomiting, pain, dyspnea, insomnia, appetite loss, constipation, diarrhea, and financial difficulties were unchanged from baseline to week 6. However, fatigue, nausea/vomiting, dyspnea, and appetite loss worsened in the chemotherapy arm.

In the KEYNOTE-355 trial, the mean values from baseline increased (worsening symptoms) in fatigue and nausea/vomiting. In contrast, the mean values were unchanged in both treatment groups’ pain, insomnia, diarrhea, and financial difficulties. Dyspnea, appetite loss, and constipation worsened in the pembrolizumab-chemotherapy arm, while they remained unchanged in the placebo-chemotherapy arm.

In the ASCENT trial, statistically significant superiority and clinically meaningful differences were reported for SG vs. TPC in fatigue, pain, dyspnea, and insomnia. However, SG was reported to be inferior to TPC in nausea/vomiting and diarrhea, though the difference in nausea/vomiting was not statistically significant. Furthermore, higher proportions of patients in the SG arm had grade 3/4 adverse events, including neutropenia and diarrhea.

In the Olaparib arm, improvements were reported in fatigue, pain, dyspnea, insomnia, appetite loss, and diarrhea across all visits compared to chemotherapy. However, nausea/vomiting was reported to be better in the chemotherapy arm compared to Olaparib.

In the EMBRACA trial, statistically significant differences favoring Talazoparib were reported in fatigue, pain, insomnia, and appetite loss. However, no statistically significant differences were observed in nausea/vomiting, dyspnea, constipation, and diarrhea (Table S4).

##### Time to Deterioration (TTD)

In the IMpassion130 trial, no clinically meaningful deterioration was observed.

In the KEYNOTE-119 trial, the median TTD for nausea/vomiting was 7.7 months and 10.4 months for diarrhea in the pembrolizumab arm.

In the ASCENT trial, the median TTD was longer in fatigue (SG vs. TPC: 7.7 weeks vs. 6.0 weeks, HR = 0.82, 95% CI 0.66–1.00, *p* < 0.05) and pain (SG vs. TPC: 21.6 weeks vs. 9.9 weeks, HR = 0.60, 95% CI 0.48–0.74, *p* < 0.001). Additionally, the median TTD in SG was longer than TPC in dyspnea, insomnia, and financial difficulties. However, higher proportions of patients in the SG arm showed clinically meaningful deterioration in diarrhea (statistically significant) and nausea/vomiting (difference not significant).

In the EMBRACA trial, significantly delayed TTD was observed in all symptoms, with a significantly greater delay in pain symptoms in the Talazoparib arm (median TTD 22.7 months vs. 7.5 months in the chemotherapy arm, HR = 0.34, 95% CI 0.22–0.50, *p* < 0.0001), and in fatigue (median TTD 17.1 months vs. 7.1 months, HR = 0.40, 95% CI 0.28–0.56, *p* < 0.0001). For nausea/vomiting, median TTD was not reached in the Talazoparib arm vs. 9.9 months in the chemotherapy arm (HR = 0.42, 95% CI 0.27–0.64, *p* < 0.0001) (Table S5).

##### Time to Improvement (TTI)

In the ASCENT trial, SG showed significantly shorter TTI in physical functioning, pain, and dyspnea.

In the EMBRACA trial, statistically significant improvement from baseline was observed in the Talazoparib arm in fatigue (‒3.9, 95% CI ‒6.2 to ‒1.6), pain (‒7.5, 95% CI ‒10.0 to ‒5.1), insomnia (‒7.1, 95% CI ‒9.5 to ‒4.7), appetite loss (‒5.1, 95% CI ‒7.9 to ‒2.4), and constipation (‒3.3, 95% CI ‒5.9 to ‒0.8).

The summarized results are shown in Table 4. Detailed domain-specific quality-of-life outcomes are provided in the Supplementary File.

## 4. Discussion

Our study reported patient-reported outcomes from multiple trials of novel therapies for triple-negative breast cancer (TNBC) across early and advanced disease. These trials evaluated immunotherapies (the PD-1 inhibitor pembrolizumab and the PD-L1 inhibitor atezolizumab), the Trop-2-targeted antibody–drug conjugate Sacituzumab govitecan, and PARP inhibitors (Olaparib, Talazoparib). Immune checkpoint inhibitors like pembrolizumab and atezolizumab work by unleashing anti-tumor T-cell responses and have gained approval in TNBC—pembrolizumab for PD-L1–positive metastatic TNBC and for high-risk early-stage disease [22,23], and atezolizumab (with nab-paclitaxel) as the first checkpoint inhibitor approved for advanced TNBC with PD-L1 expression [24].

Sacituzumab govitecan is a novel antibody–drug conjugate that delivers SN-28, an active metabolite of irinotecan and an inhibitor of topoisomerase I, to Trop–2-expressing TNBC cells; it has been shown to prolong progression-free and overall survival in refractory metastatic TNBC [25]. Similarly, PARP inhibitors such as Olaparib and Talazoparib target deficient DNA repair in germline-associated BRCA1/2-mutated, HER2-negative metastatic disease [26].

### 4.1. Completion and Compliance Rates

Across the trials, PRO assessment completion and compliance rates were high at baseline (often >90%) but tended to decline over time, which impacted the interpretation of results. In early-stage TNBC, for example, the KEYNOTE-522 trial (neoadjuvant/adjuvant pembrolizumab) reported initial PRO completion above 90%, followed by a marked drop to about 60–80% by mid-neoadjuvant therapy (around week 21). This attrition meant that the later adjuvant-phase PRO data (beyond ~24 weeks of therapy) were limited and primarily reflected patients who remained on pembrolizumab, potentially underrepresenting those who discontinued early. In metastatic trials, attrition was even more pronounced. In the OlympiAD study of Olaparib, PRO completion fell to roughly 47% by week 36. The EMBRACA trial (Talazoparib) similarly saw diminishing numbers of patients over time—by week 36, only 13.2% of patients in the chemotherapy arm remained on study (vs. 37.3% in the Talazoparib arm). Such differential dropout (with fewer patients remaining in the control chemotherapy groups) can bias QoL comparisons since the sickest patients often discontinue therapy (and stop reporting) earlier.

Notably, despite declining completion rates, compliance among patients still participating at each time point was generally high in all trials (often >80–90% of expected questionnaires returned). This suggests that PRO data are reliable for those who remained on study, but the missing data from those who dropped out must be considered when interpreting overall quality-of-life trends. High PRO compliance in studies like IMpassion031 (atezolizumab in early TNBC, with >85% PRO completion even after treatment discontinuation) demonstrates that diligent follow-up is possible. However, when compliance falters, it constrains confidence in late-term results.

### 4.2. Quality of Life in Early-Stage vs. Metastatic Settings

The impact of these novel treatments on patient quality of life (QoL) must be interpreted in the context of cancer stage and treatment intent. In early-stage TNBC (curative intent), adding immunotherapy to chemotherapy did not dramatically worsen overall QoL compared to chemotherapy alone, aside from expected transient declines during the neoadjuvant period. The IMpassion031 (atezolizumab) and KEYNOTE-522 (pembrolizumab) trials reported that global health status and functioning scores dropped during neoadjuvant chemotherapy in both the immunotherapy and control arms. This reflects the acute toll of multi-agent chemotherapy administered in both arms. For instance, IMpassion031 noted a clinically meaningful decline in global QoL from Cycle 3 to 5 of neoadjuvant therapy, which then improved by Cycle 6 (end of neoadjuvant therapy). Similarly, in KEYNOTE-522, global QoL scores decreased from baseline to the end of neoadjuvant therapy in both the pembrolizumab-chemotherapy and chemotherapy-alone groups.

Importantly, after surgery, during adjuvant therapy and follow-up, QoL in these early-stage trials rebounded. By 24 weeks into adjuvant treatment, global QoL and functioning in KEYNOTE-522 had improved toward baseline levels in both arms, with no significant between-group difference observed (LS mean difference in global score ~ –0.4 points). These findings suggest that while the addition of checkpoint inhibitors may intensify short-term side effects (as indicated by slightly greater early declines in some functioning domains with pembrolizumab), survivors of the neoadjuvant regimen recover similarly to those who received chemotherapy alone. In other words, immunotherapy did not impose a lasting QoL decline in the curative setting. Patients’ physical and role functioning did deteriorate during neoadjuvant therapy—IMpassion031 observed clinically meaningful drops in these domains by cycle 2–3 of treatment—but by the completion of adjuvant therapy, physical, role, and emotional functioning had largely stabilized or returned to baseline in both arms. Emotional well-being was reported to remain stable throughout treatment in IMpassion031. While KEYNOTE-522 noted small decreases in emotional and cognitive functioning during neoadjuvant chemotherapy for both arms, these differences were minor and equal between treatment groups. Taken together, early-stage trials indicate that incorporating pembrolizumab or atezolizumab into standard chemotherapy does not significantly compromise health-related QoL beyond the transient effects of chemotherapy itself, and any added acute toxicity is temporary and manageable within the treatment period.

In the metastatic setting (palliative intent), maintaining QoL is the goal, and it is here that the novel agents showed clear advantages over chemotherapy. Patients with advanced TNBC often begin treatment with an impaired baseline QoL (in these trials, baseline global QoL scores were in the mid-60s, lower than in early-stage patients). Trials of immunotherapy in advanced TNBC suggest that checkpoint inhibitors can preserve QoL as well as chemotherapy—and possibly better for certain subsets—while controlling disease. In the first-line IMpassion130 trial, which added atezolizumab to nab-paclitaxel in metastatic TNBC, overall QoL trajectories largely overlapped between the two arms during treatment. From approximately cycle 12 onward, atezolizumab-treated patients reported slightly higher (better) global health scores than those on chemotherapy alone, a trend observed in both the intent-to-treat population and PD-L1 the PD-L1-positive subgroup. Though these differences were modest, they indicate that extended therapy with atezolizumab did not erode QoL and may have some late-term QoL benefits as patients remained on treatment longer.

Pembrolizumab in advanced disease showed a similar trend. In the KEYNOTE-355 first-line trial, global QoL deteriorated slightly over 15 weeks in both arms, but the decline was less severe with pembrolizumab-chemotherapy (–2.7 points) than with chemotherapy alone. The between-group difference (~1.8 points favoring the pembrolizumab arm) was not statistically significant, implying a QoL outcome equivalent to standard chemotherapy. However, in a later-line setting (second-line or beyond) where pembrolizumab was given without concurrent chemotherapy, a more noticeable QoL benefit emerged: the KEYNOTE-119 trial reported that PD-L1–positive patients on pembrolizumab monotherapy had essentially no change in global QoL at 6 weeks (–0.5 points) versus a significant decline (–4.7 points) on single-agent chemotherapy. This suggests that immunotherapy alone, when effective, can spare patients the deterioration in well-being that typically accompanies palliative chemotherapy. However, these findings should be interpreted with caution due to the high dropout rate in this study. During the blinded treatment periods of these trials, no large or clinically meaningful differences in QoL were observed between arms—any advantage of immunotherapy was generally within the range of a few points on QoL scales.

Another consideration is what happens after treatment discontinuation. The IMpassion130 PRO analysis found that once patients went off protocol therapy (due to progression or toxicity), the atezolizumab and placebo arms experienced declines in QoL during follow-up. This likely reflects the impact of disease progression and second-line treatments rather than the study drugs. In other words, as long as patients’ disease was controlled on first-line therapy, their QoL remained stable; once the cancer progressed, QoL worsened irrespective of initial assignment. This shows that on-treatment QoL captured the tolerability of the therapy, whereas post-progression QoL reflected the broader disease course and subsequent treatments. Overall, the advanced disease trials demonstrated that novel agents such as pembrolizumab and atezolizumab can maintain QoL relative to standard care, and any small early differences tended to favor the investigational arm, especially in subsets most likely to respond (e.g., PD-L1–positive tumors).

Thus, the clinical value of QoL outcomes must be stage-specific: even modest improvements in advanced settings can meaningfully reduce symptom burden and enhance daily life, while in early-stage settings the emphasis is on balancing transient treatment-related declines with long-term curative benefit. These findings are summarized in Table 4, which integrates treatment arms, study design, and overall QoL effects.

### 4.3. Quality of Life Domains and Time to Deterioration

Across both early and metastatic settings, physical and role functioning were the domains most affected by active treatment, reflecting the side effects and functional limitations induced by chemotherapy (and combined modalities). In the neoadjuvant immunotherapy trials, patients experienced noticeable drops in physical function by the second or third cycle of therapy and in role function (ability to perform work or daily activities) by about week 2–3 of treatment. These declines were observed in both immunotherapy and control arms. However, some analyses showed a slightly greater magnitude of worsening when pembrolizumab or atezolizumab was added—for example, KEYNOTE-522 reported a more pronounced negative impact on physical and role functioning scores in the pembrolizumab–chemotherapy arm than in the chemotherapy-alone arm during the neoadjuvant phase. This suggests that combining chemotherapy with immunotherapy can intensify certain side effects (e.g., fatigue, which would manifest as lower physical/role function). Reassuringly, these functional domains tended to rebound after the acute treatment phase. By the adjuvant phase in early-stage trials, physical function had stabilized, and role function improved toward baseline.

In the metastatic trials, baseline physical functioning was lower (often in the 70s) due to advanced disease, and subsequent changes depended on treatment efficacy. Patients receiving effective therapies often maintained or improved their functioning due to symptom relief and disease control, whereas those on less effective regimens saw deterioration. This was shown in the ASCENT trial: Sacituzumab govitecan improved survival outcomes and led to better scores in key functional domains compared to standard chemotherapy. Patients on Sacituzumab reported higher physical and role functioning, alongside less pain and fatigue, than those on treatment of the physician’s choice. The improvements in pain and fatigue likely reflect better tumor control (less cancer-related pain, less treatment-induced fatigue) with the ADC. In contrast, the chemotherapy group experienced worsening symptoms as the disease progressed on less effective therapies. Conversely, side-effect-specific domains highlighted the expected trade-offs: Sacituzumab was associated with more nausea, vomiting, and diarrhea than the chemotherapy arm, consistent with its known toxicity profile. Aside from these gastrointestinal symptoms, Sacituzumab was non-inferior to chemotherapy in all other assessed QoL domains, meaning it did not worsen patients’ emotional, social, or cognitive well-being.

PARP inhibitors showed domain-specific advantages over chemotherapy. In OlympiAD, nearly every QoL domain either favored Olaparib or showed no difference, while nausea/vomiting was slightly worse with Olaparib (an expected side effect of oral PARP inhibition). However, Olaparib patients had better overall QoL, where one-third of patients on Olaparib reported improvement in global health status, compared to only 13% on chemotherapy. Talazoparib in EMBRACA also led to improved or preserved functioning across domains; for instance, while chemotherapy recipients saw declines in functioning and worse symptoms over time, Talazoparib recipients had significantly better global QoL and sustained functioning with treatment. Time-to-deterioration (TTD) analyses reinforced these observations by accounting for the timing of clinically meaningful QoL decline and censoring patients who have not deteriorated. These novel agents generally delayed QoL deterioration compared to chemotherapy in the metastatic stages.

In the ASCENT trial, the median time to a clinically meaningful worsening of QoL was significantly longer with Sacituzumab (about 22.1 weeks) than with chemotherapy (12.1 weeks; HR ~0.61, *p* < 0.001). In other words, patients on Sacituzumab went longer before experiencing a drop in their QoL, consistent with better disease control. Moreover, OlympiAD reported a hazard ratio of 0.44 for time to QoL deterioration, favoring Olaparib (median not reached with Olaparib vs. ~15 months with chemotherapy), and EMBRACA showed a markedly prolonged TTD with Talazoparib (median ~20.5 months vs. 5.6 months with chemotherapy). These delays (of many months) highlight that the patient on targeted therapy remained stable in their QoL for much longer. In contrast, those on chemotherapy experienced QoL decline relatively early as the disease progressed or side effects accumulated. By contrast, in the early-stage setting, TTD was not a discriminating endpoint—for example, in KEYNOTE-522, median time to QoL deterioration was roughly similar between arms (around 5–6 months in both), reflecting that almost all patients experienced some QoL drop during neoadjuvant chemotherapy, and most recovered after initial treatment regardless of arm. Thus, the greatest QoL advantages of new therapies manifested in the metastatic context, where these agents maintained QoL over time, whereas traditional chemotherapy caused a steady decline.

### 4.4. Influence of Timing and Data Completeness

The interpretation of these results must account for when QoL was measured and who was still reporting. During active therapy (especially if blinded), QoL differences between arms are more directly attributable to treatment tolerability, whereas subsequent events may confound measurements taken after treatment discontinuation. For instance, during treatment, the IMpassion130 trial noted no meaningful QoL difference between atezolizumab and placebo. However, after stopping first-line therapy, patients in both arms showed a deteriorating QoL as their cancer progressed and second-line treatments began. This illustrates that post-progression QoL reflects disease burden and salvage therapy toxicities rather than the randomized treatment effect. Therefore, analyses confined to the on-treatment period are most appropriate for judging the tolerability of the intervention.

Additionally, data completeness issues due to patient dropout (especially in metastatic trials) possibly influenced outcomes, especially if many patients in the control arm dropped out early due to progression; the remaining respondents might be those with unusually good outcomes, potentially skewing the average QoL of that group upward (“survivor bias”). In EMBRACA, by 36 weeks, the chemotherapy arm’s PRO data came from 19 patients (13% of the initial cohort)—likely those with stable disease or slower progression. In contrast, the Talazoparib arm still had 107 patients reporting (37% of its cohort). This disparity means that direct comparisons of raw mean QoL scores at late time points could be misleading. The trials mitigated this using longitudinal mixed-effects models and TTD analyses to handle missing data and censoring. Still, the interpretation of late-term QoL outcomes should be cautious. A lack of difference at a late time point could mean true equivalence or that the sickest patients (who might have had the worst QoL) are no longer represented in the data.

However, these studies—in different populations and with different instruments—indicate that therapies like pembrolizumab, atezolizumab, Sacituzumab govitecan, and PARP inhibitors do not worsen patient-reported QoL (and in many cases improve it relative to chemotherapy), suggesting that the clinical benefits of these agents (higher pathological complete response rates, longer progression-free survival, etc.) are achieved without sacrificing the patient’s subjective well-being. Indeed, patients reported better functioning and slower deterioration with agents like Sacituzumab and the PARP inhibitors.

In summary, early-stage TNBC trials showed transient QoL decreases during treatment, with full recovery post-therapy and no lasting decline. In advanced TNBC, Sacituzumab govitecan (SG), Talazoparib, and Olaparib demonstrated improvements in QoL. SG and Talazoparib showed significant gains across various domains in the ASCENT and EMBRACA trials. However, high dropout rates and the inclusion of patients who had previously received neoadjuvant chemotherapy may impact the interpretation of these findings. Attrition and treatment history could bias the reported QoL outcomes, limiting their reflection of therapy effectiveness or side effects.

We recommend that clinical trials continue to explore the potential of immunotherapy and targeted agents in combination with other therapeutic modalities, such as chemotherapy, in various stages of the disease, with extended follow-up beyond treatment cessation to understand the long-term effects of these therapies. Additionally, implementing strategies to enhance patient retention, such as more flexible follow-up schedules and alternative data collection methods, would ensure the reliability and completeness of QoL data. For studies combining chemotherapy with novel therapies, factorial designs with subgroup analyses would help discern the interaction between the treatment groups.

## 5. Conclusions

Patients with advanced or metastatic TNBC, adjuvant antibody–drug conjugates (such as Sacituzumab-govitecan) and PARP inhibitors (like Talazoparib or Olaparib) show improvements in quality of life when compared to chemotherapy. These therapies improve survival outcomes and help maintain or improve patient well-being, offering a balance between survivability and better quality of life in the metastatic setting.

However, patients with early-stage triple-negative breast cancer (TNBC), where the treatment intent is curative, do not experience significant benefits in quality of life with the use of immunotherapy when compared to chemotherapy. Therefore, while immunotherapy may improve survival, its impact on patient-reported outcomes (PROs) in the early-stage setting is minimal, with no substantial difference in overall quality of life compared to chemotherapy.

## Figures and Tables

**Table 1:** Summary of selected studies.

Trial	Population Size		Year	Clinical Trial	Novel Therapies	Peer-Reviewed and Published	Validated Tools	Location
	Novel Therapy	Chemotherapy						
IMpassion 031	161	167	2022	✔	✔	✔	✔	Worldwide
KEYNOTE 522	762	383	2024	✔	✔	✔	✔	Worldwide
IMpassion 130	403	397	2020	✔	✔	✔	✔	Worldwide
KEYNOTE 119	94	93	2023	✔	✔	✔	✔	Worldwide
KEYNOTE 355	217	100	2023	✔	✔	✔	✔	Worldwide
ASCENT	236	183	2023	✔	✔	✔	✔	Worldwide
OlympiAD	205	97	2019	✔	✔	✔	✔	Worldwide
EMBRACA	287	144	2018	✔	✔	✔	✔	Worldwide

**Table 2:** Table showing the Treatment arms, patient population, target receptors and QoL tools used in the studies.

Trial	Year	Treatment Arms	Population	Tool	Targeted Receptors
IMpassion031	2022	Atezolizumab+ nab-paclitaxel (161)	Early-stage TNBC	EORTC QLQ-C30, FACT-G	PD-L1
		Placebo+ nab-paclitaxel (167)			
KEYNOTE522	2024	Pembrolizumab+ chemotherapy (neoadjuvant+ adjuvant) (762)	Early-stage TNBC	EORTC QLQ-C30, QLQ-BR23	PD-1
		Placebo+ chemotherapy (383)			
IMpassion130	2020	Atezolizumab+ chemotherapy (403)	Metastatic triple-negative breast cancer (mTNBC)	EORTC QLQ-C30, QLQ-BR23	PD-L1
		Placebo+ chemotherapy (397)			
KEYNOTE119	2023	Pembrolizumab (94 ^1^)	Metastatic TNBC	EORTC QLQ-C30, QLQ-BR23, EQ-5D-3L	PD-1
		Chemotherapy (physician’s choice) (93 *)			
KEYNOTE355	2023	Pembrolizumab+ chemotherapy (217)	Advanced TNBC (locally recurrent or metastatic)	EORTC QLQ-C30, QLQ-BR23, EQ-5D-3L	PD-L1
		Placebo+ chemotherapy (100)			
Sacituzumab govitecan (ASCENT)	2023	Sacituzumab govitecan (236)	Refractory/relapsed metastatic TNBC	EORTC QLQ-C30	Trop-2
		Chemotherapy (physician’s choice) (183)			
Olaparib (OlympiAD)	2019	Olaparib (205)	Germline BRCA-mutant HER2-negative mBC	EORTC QLQ-C30	PARP
		Chemotherapy (physician’s choice) (97)			
Talazoparib (EMBRACA)	2018	Talazoparib (287)	Germline BRCA-mutant HER2-negative mBC	EORTC QLQ-C30, QLQ-BR23	PARP
		Chemotherapy (physician’s choice) (144)			

Germline BRCA-mutant HER2-negative mBC (Germline Breast Cancer Susceptibility Gene-mutant Human Epidermal Growth Factor Receptor 2-negative metastatic Breast Cancer); EORTC (European Organization for Research and Treatment of Cancer); QLQ-C30 (Quality of Life Questionnaire-Core 30); QLQ-BR23 (Quality of Life Questionnaire-Breast Cancer 23); EQ-5D-3L (EuroQol-5 Dimensions-3 Levels); PD-1 (Programmed Death-1); PD-L1 (Programmed Death-Ligand 1); Trop-2 (Trophoblast Cell Surface Antigen 2); and PARP (Poly (ADP-ribose) Polymerase. ^1^ Study results are focused on patients with PD L1 CPS > 10%. * Study results were based on patients with PD-L1 Combined Positive Score (CPS) > 10.

**Table 3:** Quality of life domains assessed in each study (EORTC QLQ-C30 specific).

Trial	GHS	Role	Physical	Emotional	Cognitive	Symptom Scales	TTD	TTI
Impassion031	✔	✔	✔			✔		
Impassion130	✔	✔	✔		✔	✔	✔	
KEYNOTE-119	✔					✔	✔	
KEYNOTE-355	✔		✔	✔		✔	✔	
KEYNOTE-522	✔		✔	✔		✔		
Sacituzumab govitecan (ASCENT)	✔	✔	✔	✔	✔	✔		✔
Olaparib (OlympiAD)	✔					✔	✔	
Talazoparib (EMBRACA)	✔	✔	✔	✔	✔	✔		✔

**Table 4:** Summary of QoL Outcomes by Treatment.

Trial	Population	Global QoL	Functional Domains	Symptom Burden	Reported Statistics	Overall QoL Effect
IMpassion 031	Early-stage TNBC	Clinically meaningful decline during cycles 3–5; recovery post-treatment	Declines in physical or role functioning. Recovery by adjuvant	Similar to chemo	Reported decline Cy3–5, recovery by Cy6	Neutral—transient decline, recovery by adjuvant phase
KEYNOTE 522	Early-stage TNBC	Transient decline, recovery by adjuvant	Declines in role or physical during neoadjuvant. Rebound later	Similar to chemo	LS mean difference in GHS –0.4 at 24 weeks (NS)	Neutral—declines in neoadjuvant phase, recovery by week 24
IMpassion 130	Metastatic triple-negative breast cancer (mTNBC)	No significant difference vs. chemo	Neutral	Comparable to chemo	Median TTD 8.3 vs. 8.0 months, HR 0.97 (NS)	Neutral—no significant differences
KEYNOTE 119	Metastatic TNBC	No significant difference vs. chemo	Stable vs. decline in chemo	Similar to chemo	ΔGHS –0.49 vs. –4.7 at weeks 6; median TTD 4.3 months vs. 1.7 months	Neutral—longer TTD but not statistically significant
KEYNOTE 355	Advanced TNBC (locally recurrent or metastatic)	No significant difference vs. chemo	Neutral	Comparable to chemo	ΔGHS –2.7, NS; median TTD 5.8 vs. 5.6 months	Neutral—mixed results, mostly non-significant
Sacituzumab govitecan (ASCENT)	Refractory/relapsed metastatic TNBC	Improved vs. chemo	Superior in physical, role, emotional functioning	Less fatigue or pain, ↑ diarrhea	Statistically significant GHS improvement, *p* < 0.05	Positive—significant improvement in global health, functioning, and symptoms
Olaparib (OlympiAD)	Germline BRCA-mutant HER2-negative mBC	Improved vs. chemo	Delayed TTD in physical or role function	Reduced fatigue, pain	ΔGHS +7.5 (*p* = 0.0035); HRQoL TTD HR 0.44, *p* = 0.004	Positive—improved QoL and delayed deterioration
Talazoparib (EMBRACA)	Germline BRCA-mutant HER2-negative mBC	Improved vs. chemo	Better physical, emotional functioning	Less fatigue, pain, insomnia	ΔGHS +3.0 (*p* < 0.01); TTD HR 0.376, *p* < 0.0001	Positive—significant improvement and delayed deterioration

GHS (Global health status); HRQoL (Health-related quality of life); TTD (Time to deterioration); HR (Hazard ratio); LS (Least-squares mean); NS (Not statistically significant); Cy (Treatment cycle); Δ (Change from baseline).

## Supplementary Materials

The following supporting information can be downloaded at: https://www.mdpi.com/article/10.3390/cancers17203307/s1, Table S1: Summary of QoL findings from early-stage TNBC clinical trials. Table S2: QoL findings from metastatic TNBC clinical trials. Table S3: Time-to-deterioration results in QoL domains from metastatic TNBC clinical trials. Table S4: Reported findings in symptom domains from metastatic TNBC clinical trials. Table S5: Time-to-deterioration results in symptom domains from metastatic TNBC clinical trials. Table S6: Overall quality-of-life (QoL) outcomes and statistical measures from clinical trials of novel therapies in TNBC.
