# Supplementary material for: The Impact of Novel Therapies on Quality-of-Life in Triple-Negative Breast Cancer: A Systematic Review of Clinical Trials
Source: Cancers (Basel). 2025 Oct 13;17(20):3307. doi: 10.3390/cancers17203307 (PMC12564080; doi:10.3390/cancers17203307)
Supplement: Supplementary file 1 [file cancers-17-03307-s001.zip › cancers-3838171-supplementary.pdf]

This file contains additional tables and figures referenced in the manuscript titled:
"The Impact of Novel Therapies on Quality-of-Life in Triple-Negative Breast Cancer: A Systematic Review of Clinical Trials" by **Banice Kamau, Maxim Shulimovich, and Sinha Samridhi**.

The supplementary material provides detailed statistical results, domain-specific quality-of-life outcomes, and extended tables that were moved from the main text to improve readability and conciseness.

Table of Contents

1. **Table S1.** Summary of QoL findings from early-stage TNBC clinical trials.
2. **Table S2.** QoL findings from metastatic TNBC clinical trials.
3. **Table S3.** Time-to-deterioration results in QoL domains from metastatic TNBC clinical trials.
4. **Table S4.** Reported findings in symptom domains from metastatic TNBC clinical trials.
5. **Table S5.** Time-to-deterioration results in symptom domains from metastatic TNBC clinical trials.
6. **Table S6.** Overall quality-of-life (QoL) outcomes and statistical measures from clinical trials of novel therapies in TNBC.

Table S1. Summary of QoL findings from early-stage TNBC clinical trials.

**QoL and Symptom Domains**	**Impassion031**	**Keynote522**
GHS	Decline (Neo) [2]	Decline (Neo) [2]
Physical	Decline (Neo)	Decline (Neo) [2]
Role	Decline (Neo) [1]	Decline (Neo) [2]
Fatigue	Decline (Neo)	Decline (Neo)
N/V	Decline (Neo) [2]	Decline (Neo)
Pain	Decline (Neo) [2]	Decline (Neo) [2]
Diarrhea	Decline (Neo) [2]	Decline (Neo) [3]

Neo – Neoadjuvant phase. [1] Declined earlier. [2] Improved in Adjuvant. [3] Declined in Adjuvant

Table S2. Reported findings in QoL domains from metastatic TNBC clinical trials.

**QoL Domains**	**IMpassion 130**	**KEYNOTE 119**	**KEYNOTE 355**	**SG**	**Olaparib**	**Talazoparib**
GHS	Decline (F/U)	Decline *	Decline	Improved	Improved [s]	Improved [s]
Physical	Decline (F/U)	Decline *	Decline	Improved	Improved	Improved [s]
Role	Decline (F/U)	-	Decline	Decline	Improved	Unchanged
Cognitive	Decline (F/U)	Unchanged *	Decline	-	Improved	Unchanged

F/U – Follow Up period, *High dropout rate, [s] Statistically Significant

Table S3. Time to deterioration results in QoL domains from metastatic TNBC clinical trials.

**QoL Domains**	**IMpassion 130**	**KEYNOTE 119**	**KEYNOTE 355**	**SG**	**Olaparib**	**Talazoparib**
GHS	8	4*	5	3	15	24
Physical	6	4*	5	5	13	28
Role	6	-	-	3	22	20
Cognitive	9	-	-	-	17	26

Reported in months. *High dropout rate

Table S4. Reported findings in symptom domains from metastatic TNBC clinical trials.

Symptom Domains	IMpassion 130	KEYNOTE 119	KEYNOTE 355	SG	Olaparib	Talazoparib
Fatigue	Decline	Unchanged *	Decline	Improved [s]	Improved	Improved [s 1]
N/V	Decline	Unchanged *	Decline	Decline	Unchanged	Unchanged
Pain	-	Unchanged *	Unchanged	Improved [s 1]	Improved	Improved [s 1]
Diarrhea	Decline	Unchanged *	Unchanged	Decline	Improved	Unchanged

*High dropout rate, [s] Statistically Significant, [1] Improvement (TTI) - >10-point decrease from baseline

Table S5. Time to deterioration results in symptom domains from metastatic TNBC clinical trials.

Symptom domains	IMpassion 130	KEYNOTE 119	KEYNOTE 355	SG	Olaparib	Talazoparib
Fatigue	-	8 *	-	2	12	17
N/V	-	-	-	-	17	24 [1]
Pain	-	-	-	5	24 [1]	23
Diarrhea	-	10 *	-	-	24 [1]	-

* High dropout rate, [1] Censored

Table S6. Overall quality-of-life (QoL) outcomes and statistical measures from clinical trials of novel therapies in TNBC.

Trial	Treatment Arms	Key Findings/Statistics	Overall Effect
IMpassion031	Atezolizumab + chemo vs placebo + chemo	Decline during neoadjuvant; recovery by adjuvant. Compliance >85%	Neutral
KEYNOTE-522	Pembrolizumab + chemo vs placebo + chemo	LS mean difference in GHS: –0.41 (95% CI –2.60 to 1.77). No significant difference	Neutral
IMpassion130	Atezolizumab + chemo vs placebo + chemo	TTD in GHS: 8.3 vs 8.0 months (HR 0.97; p=0.77). No statistically significant differences	Neutral
KEYNOTE-119	Pembrolizumab vs chemotherapy	Median TTD GHS: 4.3 vs 1.7 months. Mean change –0.49 vs –4.70	Neutral
KEYNOTE-355	Pembrolizumab + chemo vs placebo + chemo	Mean change GHS: –2.69 (95% CI –5.86 to 0.48). Mostly non-significant results	Positive
ASCENT	Sacituzumab govitecan vs chemotherapy	Significant improvements in GHS, physical function, fatigue, and pain. TTD fatigue HR=0.82 (p<0.05)	Positive
OlympiAD	Olaparib vs chemotherapy	Mean GHS improvement: +7.5 (95% CI 2.48–12.44; p=0.0035). Sustained over time	Positive
EMBRACA	Talazoparib vs chemotherapy	Delay in TTD: 24.3 vs 6.3 months (HR=0.376; p<0.0001). Significant improvements across domains	Positive
